# “Eyes Open – Eyes Closed” EEG/fMRI data set including dedicated “Carbon Wire Loop” motion detection channels

**DOI:** 10.1016/j.dib.2016.03.001

**Published:** 2016-03-09

**Authors:** Johan van der Meer, André Pampel, Eus van Someren, Jennifer Ramautar, Ysbrand van der Werf, German Gomez-Herrero, Jöran Lepsien, Lydia Hellrung, Hermann Hinrichs, Harald Möller, Martin Walter

**Affiliations:** a Clinical Affective Neuroimaging Laboratory, Otto-von-Guericke University, Magdeburg, Germany; b Department of Behavioral Neurology, Leibniz Institute for Neurobiology, Magdeburg, Germany; c Department of Neurology, Otto-von-Guericke University, Magdeburg, Germany; d Max Planck Institute for Human Cognitive and Brain Sciences, Leipzig, Germany; e Department of Sleep and Cognition, Netherlands Institute for Neuroscience, An Institute of the Royal Academy of Arts and Sciences, Amsterdam, The Netherlands; f Department of Cognition and Emotion, Netherlands Institute for Neuroscience, An Institute of the Royal Academy of Arts and Sciences, Amsterdam, The Netherlands; g Department of Integrative Neurophysiology, Center for Neurogenomics and Cognitive Research (CNCR), Neuroscience Campus Amsterdam, VU University and Medical Center, Amsterdam, The Netherlands; h Department of Medical Psychology, Center for Neurogenomics and Cognitive Research (CNCR), Neuroscience Campus Amsterdam, VU University and Medical Center, Amsterdam, The Netherlands; i Department of Psychiatry and Neuroimaging Center, Technische Universität Dresden, Dresden, Germany; j Department of Psychiatry, Otto-von-Guericke University, Magdeburg, Germany; k Department of Psychiatry, University Tübingen, Tübingen, Germany

**Keywords:** Multimodal imaging, EEG/fMRI, Data acquisition, EEG preprocessing

## Abstract

This data set contains electroencephalography (EEG) data as well as simultaneous EEG with functional magnetic resonance imaging (EEG/fMRI) data. During EEG/fMRI, the EEG cap was outfitted with a hardware-based add-on consisting of carbon-wire loops (CWL). These yielded six extra׳CWL׳ signals related to Faraday induction of these loops in the main magnetic field “*Measurement and reduction of motion and ballistocardiogram artefacts from simultaneous EEG and fMRI recordings”* (Masterton et al., 2007) [1].

In this data set, the CWL data make it possible to do a direct regression approach to deal with the BCG and specifically He artifact.

The CWL-EEG/fMRI data in this paper has been recorded on two MRI scanners with different Helium pump systems (4 subjects on a 3 T TIM Trio and 4 subjects on a 3T VERIO). Separate EEG/fMRI data sets have been recorded for the helium pump ON as well as the helium pump OFF conditions. The EEG-only data (same subjects) has been recorded for a motion artifact-free reference EEG signal outside of the scanner.

This paper also links to an EEGlab “*EEGLAB: an open source toolbox for analysis of single-trial EEG dynamics including independent component analysis”* (Delorme and Makeig, 2004) [2] plugin to perform a CWL regression approach to deal with the He pump artifact, as published in the main paper “*Carbon-wire loop based artifact correction outperforms post-processing EEG/fMRI corrections-A validation of a real-time simultaneous EEG/fMRI correction method”* (van der Meer et al., 2016) [3].

**Specifications Table**

**Table:** 

Subject area	*Physics, Data acquisition*
More specific subject area	*Multimodal imaging – EEG/fMRI*
Type of data	*Figure, links, plug-in*
How data was acquired	***EEG**: 30-channel EEG, 1x EOG, 1x ECG. Recorded simultaneously are 6 Carbon-Wire Loops signals.*
	***fMRI**: normalized EPI images, recorded with Tim TRIO and VERIO (SIEMENS) MRI Scanners.*
Data format	*Raw EEG data and MR-corrected EEG data in EEGlab׳.set׳ format, Echo-Planar Imaging (EPI) data, normalized EPI data and defaced T1 data in nifti format.*
Experimental factors	*The EEG/fMRI data has been acquired under two main conditions – with and without extra artifacts from the Helium pump.*
Experimental features	*Data contains EEG during Eyes open and Eyes closed in several settings. (i) outside the MRI, (ii) inside the MRI, without scanning, (iii) inside the MRI, during EPI scanning*
Data source location	*Magdeburg, Germany and Leipzig, Germany*
Data accessibility	*Data is within this article.*

**Value of the data**

• EEG/fMRI data has additional CWL signals, which include information regarding subtle motion in EEG data. This opens up a wide range of new exploratory analyses for EEG/fMRI.
• Allows assessment of any new/other artifact BCG correction algorithm, including the possibility to use׳MRI-free’ EEG [4].
• Can be used to test and improve novel regression-based hardware calculations.
• Can be used to disambiguate motion-related BOLD activation from true task activation in alpha-power EEG/fMRI analyses, by investigating CWL signals for signs of motion
• fMRI data can be analyzed to investigate dynamic BOLD network features of resting state EEG which can be corrected for scanner artifacts leading to data comparable to EEG only (also available).
• A very recent work by Rothlubbers et al. demonstrated an elegant software-based solution to deal with Helium pump artifact when searching for interictal epileptic discharges [5]; it would be valuable to compare this software-based method to the hardware-based CWL regression method to compare the ability to deal with the Helium pump artifact.

## 1 Data

This paper links to a data download repository for downloading the CWL-EEG/fMRI data [1] (Section 1.1), as well as a plugin for EEGlab [2] (see Section 1.2), in order to perform CWL Regression on the EEG data.

In this simultaneous CWL-EEG/fMRI data set, 30 EEG channels were sampled according to the 10-20 system with 5000 Hz using an MR-compatible EEG amplifier (BrainAmp MR Plus, Brain Products, Gilching, Germany), with the reference electrode located at FCz. The EEG also includes 1 EOG channel and 1 ECG channel. In addition to the EEG, 6 CWL channels were co-registered (4 CWL׳s sewn into the EEG cap and 2 attached to the EEG cables/leads) and sampled using an MR-compatible bipolar amplifier (BrainAmp ExG). A total of 8 subjects performed a simple eyes open-eyes closed task in 6 consecutive conditions chosen to isolate different kinds of MR-related artifacts (see Section 2.3).

## 2 Experimental design, materials and methods

### 2.1 EEG and fMRI data download repository

The original MR and EEG data are deposited with the Databases and IT Group at the Max Planck Institute for Human Cognitive and Brain Sciences, Leipzig, Germany in an XNAT archive (https://xnat.cbs.mpg.de/xnat/) under project name “**Evaluation of hardware motion sensors with respect to correction of the Helium pump artifact**” and project ID: **EEGfMRI**. Questions regarding data access may be sent to the databases group leader, Dr. Roberto Cozatl (cozatl@cbs.mpg.de).

### 2.2 Software (EEGlab plugin) download repository

The regression software is currently implemented as an EEGLAB plugin at NITRC and can be downloaded at: https://www.nitrc.org/projects/cwl_eeg_fmri

Alternatively, it can be obtained via the EEGlab plugin website under the name of cwleegfmri: http://sccn.ucsd.edu/wiki/EEGLAB_Extensions_and_plug-ins

The supplementary data of the original article contains a short guide on how to use the EEGLAB plugin with any EEG data set (see Appendix A: Supplementary Data in doi:10.1016/j.neuroimage.2015.10.064)

### 2.3 EOEC task and conditions: outside/inside MRI, wit/without scanning, He OFF and He ON

Eight healthy volunteers were prepared with MRI-compatible EEG (including CW Loops) and performed a simple eyes open/eyes closed (EOEC) task in six conditions. The EOEC task consisted of five׳eyes open׳ blocks and four׳eyes closed׳ 30-s blocks within 4 min and 30 s. The screen (either a laptop screen outside or the projector screen inside the MRI scanner) presented the instructions to keep the eyes closed or open; Black/white ׳flashes׳ helped the subject notice a change in condition when the eyes were closed.

**Fig. 1:**
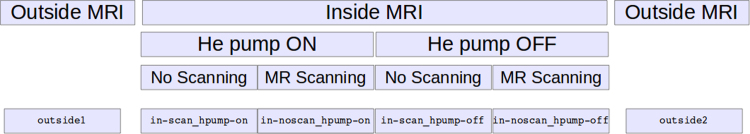
Filename convention in the EEG data set. The bottom row identifies the condition part of the EEG data file names. The conditions are: (1) outside, before going in the MRI scanner, (2) inside the MRI scanner, without scanning and the Helium pump ON, (3) inside the MRI scanner, with scanning and the Helium pump ON, (4) inside the MRI scanner, without EPI scanning and with the Helium pump OFF, (5) inside the MRI scanner, with EPI scanning and with the Helium pump OFF and finally (6) outside, after coming out of the MRI scanner. For test-retest purposes: **in-noscan** measurements were always performed before **in-scan measurements.**.

The six conditions were chosen to be able to compare MR-polluted EEG to artifact-free EEG outside of the scanner, and also to assess effects of switching on/off the Helium pump [6]. See Fig. 1 for a schematic overview and file name convention in the EEG data set.

Subjects measured at the TIM trio have data files (both EEG and fMRI) starting with **trio1**, **trio2**, **trio3** and **trio4**. Subjects measured at the VERIO system have data files (both EEG and fMRI) starting with **verio5**, **verio6**, **verio7** and **verio8**. In addition, for subjects trio1, trio3, verio5 and verio7, the helium pump ON measurement was performed *before* the helium pump OFF measurement – for subjects trio2, trio4, verio6 and verio8, this order was reversed.

Due to technical reasons, three EEG data sets are missing: For subjects trio1 and trio2, the EEG outside after the MRI session (i.e. outside2) was not measured. For subject 2, the EEG without scanning during the helium pump ON (i.e. in-noscan_hpump-on) was also not measured.

### 2.4 Markers in EEG data sets during simultaneous EEG/fMRI

The EEG data contain markers (where applicable) for (i) each MRI scan (*mri*: start of an EPI scan), (ii) R-peaks (*R*: heart beat marker), (iii) light flashes (*S 1:begin of flash*), (iv) instructions (*beo*: begin eyes open; *eeo*: end eyes open; *bec*: begin eyes closed; *eec*: end eyes closed) and (v) syncbox function (*Sync On*). R-peak markers were placed in the ECG trace using BrainVision analyzer software. Missing misplaced and double R-peak markers were controlled for. For the ‘in-scan’ condition, we also included MR-corrected EEG data. These have been artifact corrected with the Bergen EEG-fMRI Toolbox [7] according to specification mentioned in the main article [3].

### 2.5 fMRI Data

The fMRI data files are normalized EPI images (resampled to a resolution of 3×3×3 mm) and the final product of a pipeline including slice-time alignment, motion correction, and a normalization scheme involving DARTEL to use subject-specific flow-fields to directly warp the EPI images into MNI space (after coregistering the EPI data to the T1 data). The single-shot gradient-echo echo-planar imaging (EPI) sequence was equivalent for both scanners (echo time, TE, 30 ms; 192-mm field of view, 64×64 matrix, 3-mm slice thickness; 1-mm slice gap, 3×3 mm^2^ nominal in-plane resolution, 30 axial slices aligned along the AC-PC line) except for the repetition time (TR 1.95 s on the TIM Trio and 2.00 s on the Verio).

## Footnotes

Appendix A Supplementary data associated with this article can be found in the online version at 10.1016/j.dib.2016.03.001.
