# Supplementary material for: “Eyes Open – Eyes Closed” EEG/fMRI data set including dedicated “Carbon Wire Loop” motion detection channels
Source: Data Brief. 2016 Mar 9;7:990–4. doi: 10.1016/j.dib.2016.03.001 (PMC5063756; doi:10.1016/j.dib.2016.03.001)
Supplement: Supplementary file 1 — Supplementary material [file mmc1.doc]

**Conflict of Interest Statement**

The authors whose names are listed immediately below certify that they have NO affiliations with or involvement in any organization or entity with any financial interest (such as honoraria; educational grants; participation in speakers’ bureaus; membership, employment, consultancies, stock ownership, or other equity interest; and expert testimony or patent-licensing arrangements), or non-financial interest (such as personal or professional relationships, affiliations, knowledge or beliefs) in the subject matter or materials discussed in this manuscript.

Johan van der Meer

André Pampel

Eus van Someren

Jennifer Ramautar

Ysbrand van der Werf

German Gomez-Herrero

Jöran Lepsien

Lydia Hellrung

Hermann Hinrichs

Harald Möller

Martin Walter
